# Anomalous bronchus and pulmonary artery in a patient who underwent subsegmentectomy of right S6a + b

**DOI:** 10.1186/s44215-023-00053-2

**Published:** 2023-08-07

**Authors:** Yo Tsukamoto, Mitsuo Yabe, Ai Ishikawa, Daiki Kato, Takamasa Shibazaki, Takeo Nakada, Takashi Ohtsuka

**Affiliations:** Department of Surgery, The Jikei University School of Medicine, Nishishinbashi 3-19-18, Minatoku, Tokyo 105-8471 Japan

**Keywords:** Anomalous bronchus and pulmonary artery, Non-small cell lung cancer, Segmentectomy

## Abstract

This report describes the case of a woman with small-sized peripheral lung cancer who underwent thoracoscopic right superior subsegmentectomy of S6a + b. Preoperative multiplanar reconstruction computed tomography revealed an anomalous posterior basal bronchus (B10c) and pulmonary artery (A10c), branching from the right lower superior segment bronchus (B6) and artery (A6), respectively. A three-dimensional (3D) model of the bronchi and pulmonary vessels and virtual bronchoscopy images were created to confirm the anomalous bronchus and pulmonary artery. Right superior subsegmentectomy of the right S6a + b was performed with the preservation of the anomalous posterior basal bronchus and pulmonary artery, as planned preoperatively, with no intraoperative or postoperative complications. The preoperative 3D reconstruction image was useful for understanding the patient’s anatomy. Furthermore, an understanding of anatomy is necessary when performing segmentectomy in patients with anomalous bifurcations of the pulmonary artery and bronchus.

## Introduction

Segmentectomy, in which anomalies of the bronchi and pulmonary vessels are involved in the segment, is a high risk for intraoperative and postoperative complications [1–4]. Here, we present a rare case of right lobe superior segmentectomy with bronchus and pulmonary artery anomalies in the right lower superior segment.

## Case presentation

**Fig. 1:**
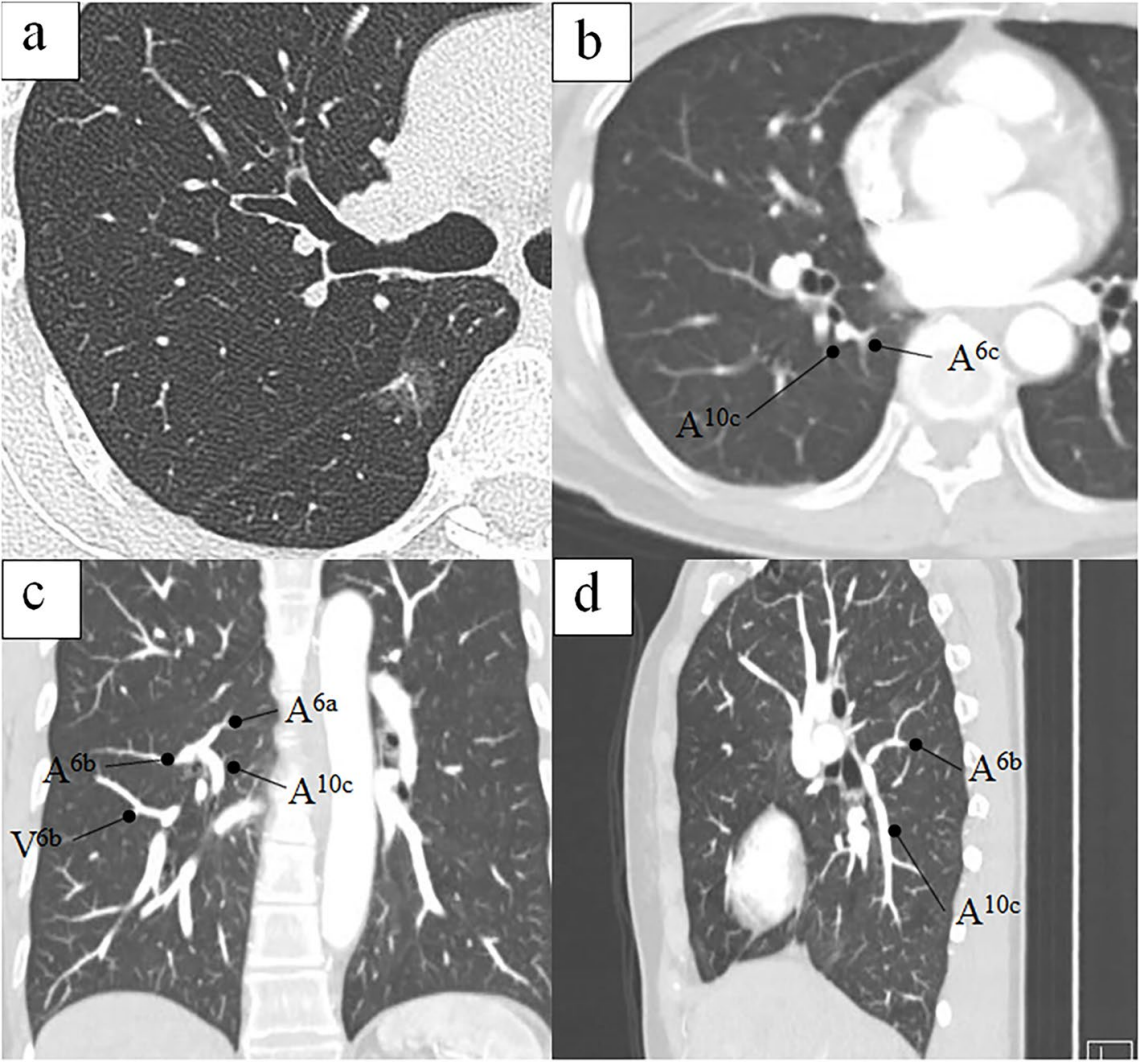
The chest computed tomography (CT) shows the tumor 14 mm in diameter and a solid component (4 mm) **a**. Preoperatively, multi-planar reconstruction CT shows an anomalous posterior basal pulmonary artery (A10c), branching from the right lower superior segment artery (A6) (**b** axial image, **c** coronal image, **d** sagittal image)

**Fig. 2:**
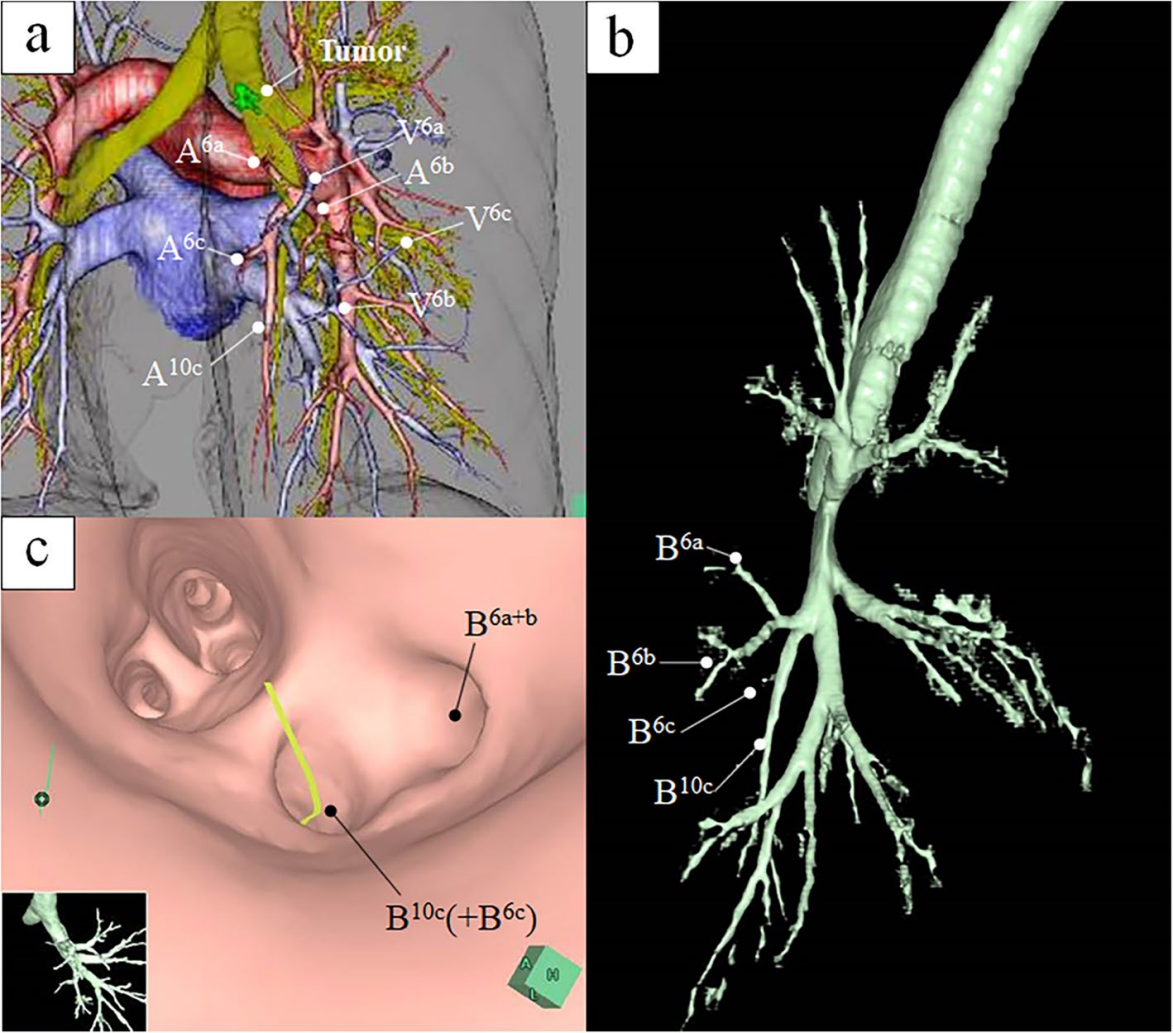
Three-dimensional model of the bronchi and pulmonary vessels (**a**, **b**) and virtual bronchoscopy images (**c**) were reconstructed using SYNAPSE VINCENT (FUJIFILM Medical Co., Ltd.). **a** A6 and A10c were a common trunk. A6c is located cranial to V6b and V6c which are intersegmental veins. **b**, **c** Anomalous posterior basal bronchus (B10c + B6c) branching from the right lower superior segment bronchus (B6a + b)

**Fig. 3:**
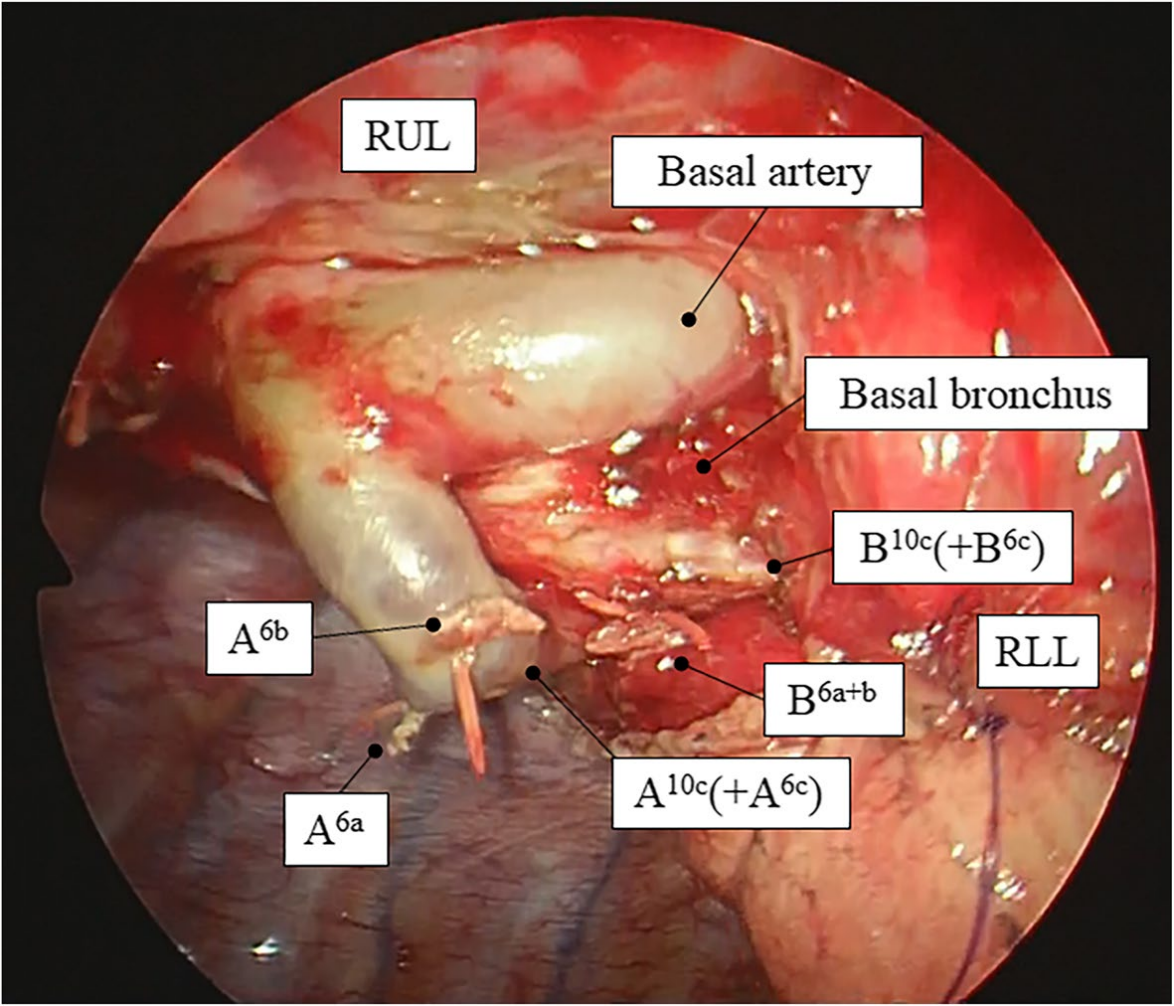
Intraoperative photo after subsegmentectomy indicated that A10c and B10c are preserved and A6a, A6b, and B6 are dissected. RUL, right upper lobe; RLL, right lower lobe

This case reports on a 59-year-old woman who was referred to our hospital because of abnormalities in the chest radiography for medical check-up. The chest computed tomography (CT) indicates pure ground-glass opacity (GGO) with a diameter of 11 mm in the right lower superior segment. The GGO was not confirmed by the chest radiography. After 1 year, the tumor had grown to 14 mm in diameter and a solid component (4 mm) was observed (Fig. 1a). Positron emission tomography and head magnetic resonance imaging were performed to confirm the absence of lymph node metastasis or distant metastasis. Since the tumor was peripherally located and its size was < 20 mm in diameter, we decided to perform a segmentectomy of the right superior segment. Preoperatively, multiplanar reconstruction CT detected an anomalous posterior basal bronchus (B10c) and pulmonary artery (A10c), branching from the right lower superior segment bronchus (B6) and artery (A6), respectively (Fig. 1b–d). A three-dimensional (3D) model of the bronchi and pulmonary vessels and virtual bronchoscopy images were reconstructed using SYNAPSE VINCENT (FUJIFILM Medical Co., Ltd.) (Fig. 2). These images further confirmed that B6c and A6c branched from the distal ends of B10c and A10c, respectively. Based on these images, we planned a right superior segmentectomy with the preservation of the bronchus and pulmonary artery in the posterior basal segment (S10). We decided to preserve S6c since it provides a sufficient margin to the tumor and branches from the distal end of B10c and A10c. Surgery was performed completely thoracoscopically with four ports. A6a and A6b were detected from the interlobar artery, each ligated proximally and transected. We confirmed, intraoperatively, B6a + b and B10c (+ B6c) using a bronchoscope, and B6a + b was ligated and dissected at the peripheral of the bifurcation (Fig. 3). Subsequently, Inflation-deflation line was confirmed by performing right lung ventilation, deflated superior segment (S6a + b) resection was performed using an automatic suture instrument. S6a + b resection was performed using 45- and 60-mm purple cartridge staplers. Since a further peripheral dissection was necessary to expose B6c and A6c, and dissecting A6a + b and B6a + b provided sufficient margin from the tumor, a subsegmentectomy was performed. No manipulation was performed on the pulmonary veins involved in S6. The patient was discharged without any complications. Pathological and immunohistological findings were adenocarcinoma in situ (14 mm in diameter), pTisN0M0 Stage 0.

## Discussion

Yaginuma et al. [5] reported the incidence of the displaced bronchi was 0.76%, mostly in the right upper lobe. Although rare, anomalous bronchial and vessel bifurcations in the right lower lobe have also been reported [6, 7]. Anatomical lung resection in patients with displaced bronchi and pulmonary artery can lead to risks of broncho-vascular injury, especially in segmentectomy [1–4]. With the increased use of CT screening and the widespread use of high-resolution thin-slice CT, small-size and ground-glass opacity lung tumors are more frequently being detected. Since segmentectomy for small peripheral non-small cell lung cancer has been shown to have good clinical outcomes [8], it is expected that it will be increasingly indicated in such population of patients. When anomalous bronchi and pulmonary vessels are present in the target segment of a patient planned for segmentectomy, preoperative anatomic planning is necessary for the safe operation. Intraoperative dissection of the wrong bronchus can result in atelectasis of the segment, and misjudgment of pulmonary vessel bifurcation can result in the need for angioplasty or the risk of fatal bleeding.

In this case, preoperative CT showed anomalous bronchi and pulmonary artery bifurcation. A 3D model of the bronchi and pulmonary vessels and virtual bronchoscopy images were reconstructed to confirm the anatomic findings. The subsuperior bronchi (B ∗) was defined as a separate bronchus originating from the basal or secondary basal trunk bronchus [9, 10]. Since A10 and B6 had only two branches and the position of this bronchial segment was running towards the basal (diaphragmatic) area, but not to the lateral or posterior area,, we concluded that the bronchus and pulmonary artery extending into the basal segment was not B ∗ and A ∗ but an abnormal branch of B10 and A10. A6a and A6b were dissected as preoperatively planned, and A10c was preserved. Furthermore, the intraoperative bronchoscopy confirmed the bifurcation of B6a + b and B10c (+ B6c), while ensuring that only B6a + b was dissected. The surgery was terminated after confirming that S10 could be fully expanded. Similar to the present case, there is a previous report of a displaced right B10c bronchus originating from the superior bronchus of the lower lobe (B6) [7]; however, in that report, common trunk B10c and B6c and pulmonary artery branching were not discussed. Leaving S10 with dissection of B10c and A10c leads to pulmonary congestion and atelectasis should be avoided.

In segmentectomy, it is crucial to understand the individual patient’s anatomy preoperatively to account for the possibility of anomalous bronchial and vessel bifurcation. The 3D model, virtual bronchoscopy, and intraoperative bronchoscopy allowed for safe subsegmentectomy without intra and postoperative complications.

## Data Availability

All data generated or analyzed during this study are included in this published article.
